# Antioxidants, immunotoxicological, and histopathological impacts of polypropylene microplastics emitted from paper cups on *Clarias gariepinus* and the ameliorating role of *Spirulina* and recovery

**DOI:** 10.1007/s10695-025-01587-8

**Published:** 2025-10-22

**Authors:** Zainab Eid, Usama M. Mahmoud, Hanem S. Abdel-Tawab, Alaa El-Din H. Sayed

**Affiliations:** 1 Zoology Department, Faculty of Science, Assiut University, Assiut, 71516 Egypt; 2 Molecular Biology Research & Studies Institute, Assiut University, Assiut, 71516 Egypt

**Keywords:** Polypropylene, Fish, *Spirulina*, Immune, Antioxidants, Histopathology

## Abstract

Fish are particularly vulnerable to microplastics (MPs), especially polypropylene microplastics (PP-MPs), which are widely used and environmentally persistent. Despite their prevalence, little is known about their impact on fish immune systems. Thus, this study’s goal was to look at the antioxidants, immunotoxicological, and histopathological impact of PP-MPs on African catfish *Clarias gariepinus* and the ameliorating role of *Spirulina* and recovery. A total of 108 fish, weighing 125 ± 3 g and 27 ± 2 cm, were acclimated and divided into six experimental groups (in triplicate): control, PP-MPs-treated groups (0.14 and 0.28 mg/L), PP-MPs + *Spirulina* (200 mg/L), and *Spirulina* alone. Fish were exposed to treatments for 15 days, followed by a 45-day recovery period. Antioxidant enzymes (SOD, CAT, GST, MAD), immune biomarkers, and histopathological changes in the spleen and head kidney were assessed. PP-MPs exposure led to a significant (*p* < .05) decline in antioxidant enzymes and immune biomarkers compared to controls, with increased melanomacrophage centers and tissue damage. *Spirulina* supplementation significantly improved immune and antioxidant responses, although some parameters, like MAD and histopathological alterations, showed incomplete recovery even after 45 days. PP-MPs have immunotoxic and oxidative effects on *Clarias gariepinus*, with partial recovery possible through *Spirulina* supplementation. However, full restoration of immune tissue morphology requires longer recovery periods. The observed immune alterations were closely associated with histopathological damage in key immune organs.

## Introduction

Plastic items are inexpensive and convenient, which makes them widely used (Li et al. 2020). However, when used and disposed of improperly, they can harm the environment significantly (Laskar and Kumar 2019). In aquatic environments, PP-MPs, a widely used plastic in everyday products and industrial manufacturing, are among the most prevalent and severely polluting types of plastic (Yan et al. 2024). According to reports, the percentage of other MPs like polystyrene (PS) and polyethylene (PE) is said to be reduced when the size of PP-MPs plastic (less than 1 mm) is smaller (Li et al. 2021a). Due to its poor resistance to oxidation, ultraviolet (UV) radiation, and temperature fluctuations, as well as its vulnerability to hydrolysis and other biotic and abiotic factors such as bacterial degradation, UV exposure, and thermal breakdown, PP-MPs degrade rapidly in ocean environments and easily fragment into smaller particles. These drawbacks of PP-MPs are detailed by Zhang et al. (2020). The following could be the cause of MPs joining forces with viruses to make hosts more vulnerable: The physical makeup of MPs damages fish’s internal organs, making it harder for fish to fend off viruses (Beans 2023; Sangkham et al. 2022; Seeley et al. 2023); the virus potentially adsorbs on MPs, extending its infectiousness (Shan et al. 2023); MPs modify the host’s immune response by inhibiting cells from generating proteins that may stop the virus from spreading (Beans 2023; González-Fernández and Cuesta 2022; Wang et al. 2023, 2021); fish may be endangered by poisonous substances present in plastics, such as flame retardants, which can weaken their immune systems and reduce their ability to fight off viruses (Beans 2023).

The aquatic ecosystem is unstable and frequently subjected to different environmental conditions. Hydrobionts adapt by altering how the body’s internal systems operate in response to the continuing changes (Suvorova et al. 2023). MPs’ small size makes them easy for aquatic species to consume, making them resemble food particles (Kögel et al. 2020; Rainieri et al. 2018). While some MPs that are consumed are eliminated through feces, others enter the tissues and cause harm to organs (liver disease, acinar epithelial necrosis in the pancreas, lipidosis, triggering cell death, enhancing coughing behavior, decreasing predatory behavior, enhancing mucus cell secretory capacity, triggering physiological alterations, et cetera) (Hu et al. 2022; Lee et al. 2023; Liang et al. 2023; Revel et al. 2020; Wang et al. 2020; Zhang et al. 2023). As stated by Dalmo et al. (1997) and Mokhtar et al. (2023), the immune system of fish primarily consists of immunological tissues, immune cells, and various immune factors. One effect of ingesting MPs may be immune system modification, which could affect an animal’s defense and general health (Espinosa et al. 2018). Simultaneously, the immune system may be impacted chemically (by potentially toxic substances that MPs may include, absorb, or discharge) (Rochman et al. 2013) or physically (by physically obstructing the digestive organs, this lessens the absorption of nutrients and causes problems with energy allocation) (Cole et al. 2013).

The innate immune response in fish serves as their primary defense against invasive infections and consists of several immunological defense mechanisms (Magor and Magor 2001). A fish’s innate immunity is made up of humoral factors, natural killer cells, anatomical barriers, and phagocytes (granulocytes and monocyte-macrophages). Building an effective adaptive immune response requires the last group (Bhattacharya et al. 2010; Espinosa et al. 2018). A network of splenic ellipsoids, lymphoid tissue, and MMCs make up the spleen and these structures aid in phagocytosis and antigen absorption (Ellis and Smith 1966). Up until maturity, the spleen and head kidney perform hematopoiesis comparable to that of the bone marrow (Wolber et al. 2002). The spleen is responsible for the production of antibodies as well as the breakdown and processing of antigens (Dalmo et al. 1997). Furthermore, according to Graf and Schlüns (1979), the spleen is a significant hematopoietic organ that produces melanin macrophages, endothelial cells, reticulocytes, and blood cells.

According to Fishelson (2006), the head kidney (HK) is the principal immunocompetent organ in teleosts and head kidney macrophages (HKMs) are essential components of the fish's innate immunity. By releasing reactive oxygen species, the HKMs absorb invasive pathogens and eliminate those (Nayak et al. 2007). The principal cell types in the head kidney are macrophages, which group together to form melanomacrophage centers (MMCs), and lymphoid cells, primarily B cells, which are present throughout all embryonic stages (Uribe et al. 2011). In teleostean or “true bony” fish, the head kidney is a vital endocrine and hemopoietic lymphoid organ that is comparable to the mammalian adrenal gland because it generates and secretes a variety of hormones, including proopiomelanocortin, growth hormone, catecholamines, prolactin, melanin-concentrating hormone, and cortisol (reviewed by Gallo and Civinini (2003) and Uribe et al. (2011)). The zonal cortex and medulla of the head kidney are not visible, so it lacks the distinct anatomy of its mammalian counterpart (Geven and Klaren 2017). Rather, the tissue that produces hematopoietic antibodies and cytokines encircles and embeds cortisol-producing interregnal cells and catecholamine-producing chromaffin cells (Geven and Klaren 2017).

The head kidney is considered one of the most important immune organs in teleost fish due to its high populations of macrophages and lymphocytes, which can generate strong immunological responses (Joerink et al. 2006). Fish macrophages, in particular, release key pro-inflammatory cytokines that initiate host immune responses (Lage et al. 2006; Pressley et al. 2005). Given its central role in immunity, the head kidney serves as a valuable target tissue for investigating the immunotoxic effects of PP-MPs and the immunomodulatory properties of SP in *Clarias gariepinus*. Understanding these effects has practical implications for aquaculture, including improved fish growth, enhanced resistance to pathogens, and increased marketability of the final product (Wang et al. 2017).

A group of leukocytes known as lymphocytes is responsible for immunological surveillance and humoral and cellular immune response development (Suvorova et al. 2023). Primordial macrophages and monocytes have an elevated phagocytic activity toward the by-products of tissue and cell degradation, neutralize toxins, and participate in the cytokine generation process (Suvorova et al. 2023). Neutrophils and eosinophils play a role in the phagocytosis of microorganisms and the production of immune response mediators, contributing to non-specific immunity (Havixbeck and Barreda 2015; Scapigliati 2013). Regretfully, not much is understood about how the PP-MPs affect fish immunity. Therefore, this study aimed to investigate the potential effects of PP-MPs as a type of microplastics released from paper cups on African catfish (*C. gariepinus*), considering the ongoing rise in global plastic production and its subsequent environmental release.

One type of filamentous photoautotrophic cyanobacterium is *Spirulina* that is found in a variety of settings, including soils, lakes, and brackish and marine water, where it forms a green scum on the water’s surface (Richmond 2003). SP is the most popular algae-based food product in the USA and Asia (Hadiyanto et al. 2021). They are Food and Drug Administration (FDA)–certified and widely accepted as safe for consumption (Lucas et al. 2018). According to scientific research, microalgae, particularly *Spirulina*, are commonly used as dietary supplements because of their high protein content (> 60%), vitamins, minerals, carbs, carotenoids, xanthophyll, and γ-linolenic acid (Jaat et al. 2013). SP has been shown to have numerous beneficial effects in various fish species, including Siberian sturgeon (*Acipenser baeri*) (Palmegiano et al. 2005), common carp (*Cyprinus carpio*) (Watanuki et al. 2006), tilapia (*Oreochromis niloticus*) (Ragap et al. 2012), and African Sharptooth Catfish (*C. gariepinus*) (Sayed and Fawzy 2014). During the last decades, research has focused on using eco-friendly supplements to enhance fish development, immunity, and disease resistance (Dawood 2016; Van Doan et al. 2020; Van Hai 2015). SP has been shown to be both restorative and protective for aquatic creatures in toxicity conditions (Abdelkhalek et al. 2017; Dawood et al. 2017). *Spirulina* has been shown to boost fish yield (Sayed and Fawzy 2014) and act as an immunomodulator (Hironobu et al. 2006). Nevertheless, the possible remedial benefits of *Spirulina* against the toxicity of PP-MPs on the immune system of African catfish have not received much attention. Until now, few studies have examined the immune response of the head kidney and spleen to PP-MPs microplastics in *C. gariepinus* using a combined histology and immunology methodology. Consequently, this study’s main objective is to find out how PP-MPs affect African catfish’s leucocytes, antioxidants, immunological indices, and immunological tissues like the spleen and head kidney.

African catfish (*C. gariepinus*) is easy to rear and has good resistance to environmental stressors, making it a popular choice for customers with high market value (Abdel-Tawwab et al. 2018). In addition to being a significant food source and productive species in Egyptian and international aquaculture, the African catfish is one of the most well-known and common freshwater fish in the world (Hamed et al. 2024). Catfish are an effective ecotoxicological model for assessing the potential toxicity of substances in both natural ecosystems and experimental settings (Hamed et al. 2022; Sayed et al. 2022).

## Materials and methods

### Chemicals and *Spirulina platensis*

PP-MPs were microsized, spherical in shape, with an average diameter of approximately 35 µm, and were obtained from Sigma-Aldrich. PP-MPs were accurately weighed and pre-suspended in a measured volume of dechlorinated water. The dispersion was achieved using continuous magnetic stirring to prevent particle aggregation and ensure even distribution. The methodology for characterizing PP-MPs was carried out using scanning electron microscopy at the scanning electron microscopy unit (SEMU) in our previous research (Eid et al. 2024).

*Spirulina* tablets were bought from Japan Algae Co., Ltd.

### Specimens’ collection

African catfish (*C. gariepinus*) juveniles were taken out of the aquaponic system and placed in 100-L tanks in Assiut University’s Fish Pollution Laboratory. The tanks were filled with dechlorinated water and the fish were given 4 weeks to acclimate. Throughout the studies, the fish were maintained in controlled environments with water at 28 °C, a pH of 7.4, dissolved oxygen levels of 18.14 mg L^−1^, conductivity of 0.478 µS/cm, and a light:dark cycle of 12 h. The fish were fed a commercial pellet diet (SKRETTING Company, Egypt) at 3% of their body weight (125 ± 3 g). The diet contained 30% crude protein, 3800 kcal/kg of energy, 5.71% fiber, and 3.08% crude fat. Every day, the water was replaced in order to remove contaminants from metabolic waste. To ensure their health, the African catfish were checked both before and after acclimation according to Batts et al. (2020).

### Experimental design

Six experimental groups of catfish (125 ± 3 g and 26 ± 27 ± 2 cm) were assigned in triplicate once the fish had acclimated to the lab environment (18 fish/group, 6 fish in each replicate). The groups were exposed to PP-MPs, *Spirulina* (200 mg/L), PP-MPs (0.14 and 0.28 mg/L PP-MPs), and PP-MPs + *Spirulina* (0.14 mg/L PP-MPs + 200 mg/L SP and 0.28 mg/L PP-MPs + 200 mg/L SP) over a period of 15 days. Every fish group was kept in a separate tank with 70 L of water, it was altered by half, and redoing of the PP-MPs concentrations was diminished every 2 days. The fish were then allowed to recover for 45 days under normal conditions, with half of the water being replaced daily. The selection of PP-MP dosages was based on Lei et al. (2018) and Jemec Kokalj et al. (2022) who reported that these concentrations have already been reported in the environment, whereas the dose of *Spirulina* was chosen in accordance with Sayed and Authman (2018).

### Blood sample collection

Upon completion of the duration of exposure and recuperation, ice was used to anesthetize six fish, chosen at random from all of the groups (2 fish from each replicate per sampling period), in an effort to lessen stress (Wilson et al. 2009). Blood samples were collected from the caudal veins and placed into tubes (approximately 1 mL) with and without heparin for the analysis of leukocytes, immunological parameters, and antioxidant biomarkers.

### Leucocytes and immunological parameters analysis

Using a hemocytometer, leucocytes were counted from blood in heparinized tubes according to Stevens (1997). After allowing the blood to coagulate at 4 °C, we isolated serum from the blood in non-heparinized tubes by centrifuging it at 5000 rpm for 20 min to assess immunological parameters and antioxidant biomarkers. A turbidity assay technique was used to quantify the activity of lysozyme (LYZ) (Parry et al. 1965). Using an ELISA kit, the amounts of immunoglobulin M (IgM) in the serum samples were determined in compliance with a previously documented protocol (Overkamp et al. 1988). Using a formula previously published (Siwicki et al. 1994), the phagocytic (PA) activity (%) of leukocytes was calculated.

### Antioxidant biomarkers assay

Serum glutathione S-transferase (GST; EC 2.5.1.18) activity was measured in accordance with Habig et al. (1974) as the conjugation of 1-chloro-2,4-dinitrobenzene (CDNB) with reduced glutathione. The increase in absorbance at 340 nm, corresponding to the formation of the conjugate, was monitored spectrophotometrically. One unit of enzyme activity was defined as the amount catalyzing the conjugation of 1 µmol of CDNB with GSH per minute at 25 °C. Superoxide dismutase (SOD, EC 1.15.1.1) activity in serum was assessed using the method described by Sun et al. (1988). This assay is based on the inhibition of nitro blue tetrazolium (NBT) reduction by the superoxide radicals generated through a xanthine–xanthine oxidase system. Absorbance was measured at 560 nm, and one unit of SOD activity was defined as the amount of enzyme causing 50% inhibition of the NBT reduction. The catalase activity (CAT, EC 1.11.1.6) was assessed according to the method described by Hadwan and Abed (2016). After applying CAT inhibitor for 1 min, the reaction between the CAT and a known volume of H_2_O_2_ was stopped. A chromophore is produced when the residual H_2_O_2_ reacts with 3–5-dichloro-2-hydroxybenzene sulfonic acid and 4-aminophenazone in the presence of peroxidase, the strength of which is inversely related to the concentration of CAT in the sample. The intensity of the resulting color, measured at 510 nm, is inversely proportional to catalase activity. The malondialdehyde activity was measured based on the methodology of Li and Chow (1994). The reaction between MDA and thiobarbituric acid (TBA) under high temperature and acidic conditions produces a pink chromogen, which was measured spectrophotometrically at 532 nm.

### Histological and histochemistry assessment

The spleen and head kidney of *C. gariepinus* were dissected following exposure and recovery and preserved in a neutral formalin solution of 10%, followed by dehydration using a graded ethanol series (70%, 75%, 80%, 90%, and 100%). The specimens were embedded in paraffin at 62 °C and sectioned using a precision microtome (MT-990; RMC Products, USA) about 5 µm in thickness. Sections were stained with Harris hematoxylin and eosin (Biancotti et al. 2020), and collagenous fibers were investigated with the use of Sirius Red staining as described by Courtoy et al. (2020). The samples were examined using an Olympus BX-51 light microscope (Olympus Corp., Japan) and an ARTCAM-150 PIII digital camera.

Melanomacrophages centers (MMCs) measurements were performed on images of the spleen and head kidney tissues. To count the MMCs, six fields were chosen at random from each slide. Consequently, 60 measurements at a 400 × magnification were taken for each treatment. After photographing each field, the area was measured in square micrometers (µm^2^) using a calibrated micrometer with Motic Images Plus 2.0 software, and the number of MMCs per field was counted.

### Statistical analysis

The fundamental statistics, including means, standard errors, and ranges, were estimated. The pattern of variation among groups was analyzed using one-way analysis of variance (ANOVA) using the IBM-SPSS (2012) software at a significance level of 0.05. For multiple comparisons among groups, both Tukey’s HSD test and Dunnett’s test were employed.

## Results

At the 0.05 level of significance, values that share the same letters within a parameter do not differ (horizontal comparison).

### Leucocytes and immunological parameters

Table 1 shows the leucocytes of catfish exposed to PP-MP (0.14 mg/L), PP-MP (0.28 mg/L), PP-MP (0.14 mg/L) + SP (200 mg/L), PP-MP (0.28 mg/L) + SP (200 mg/L), and SP (200 mg/L) as a positive control for a period of 15 days. With the exception of monocytes, all parameters (WBCs, large lymphocytes, small lymphocytes, neutrophils, and eosinophils) indicated that the control group and the treated groups differed significantly (*p* < 0.05). The parameters displaying significance or non-significance either decreased with the rise of PP-MP doses from 0.0 inthe control to 0.28 mg/L (WBCs, large lymphocytes, small lymphocytes, and monocytes) or increased with such increased doses (neutrophils and eosinophils) (Table 1). All of the parameters showed non-significant (*p* > 0.05) variations between the 200 mg/L SP exposed group and the control group. Moreover, the bioremediation with 200 mg/L SP in the group exposed to 0.14 mg/L PP-MPs demonstrated a significant (*p* < 0.05) rise in WBCs, large lymphocytes, small lymphocytes, neutrophils, and eosinophils and non-significant (*p* ≥ 0.05) rise in monocytes when in contrast to the same exposed group without *Spirulina* and the control, while the bioremediation with 200 mg/L SP in the 0.28 mg/L PP-MPs exposed group revealed a significant (*p* < 0.05) rise in WBCs and small lymphocytes and non-significant (*p* ≥ 0.05) decrease in neutrophils, large lymphocytes, and monocytes when compared to the same exposed group without *Spirulina*. Following 45 days of recovery, similar trends of significant variations that pointed either upward or downward were still notable in comparison to the control group, while in comparison to the exposure period, leucocytes exhibited a non-significant (*p* ≥ 0.05) decrease in the 0.14 mg/L PP-MPs exposed group and an increase in the 0.28 mg/L PP-MPs exposed group (Table 1). The bioremediation with 200 mg/L SP in the 0.14 and 0.28 mg/L PP-MPs exposed groups following 45 days of recovery showed non-significant (*p* ≥ 0.05) reduction in comparison to the exposure period.

**Table 1:** Leucocytes and immunological parameters (mean ± SE and min–max range) of the African catfish exposed to PP-MPs (0.14 mg/L), PP-MPs (0.28 mg/L), PP-MPs (0.14 mg/L) + SP (200 mg/L), PP-MPs (0.28 mg/L) + SP (200 mg/L), and SP (200 mg/L) as a positive control for 15 days followed by a 45-day period of recovery

Treatments	Control	0.14 mg/L PP-MPs	0.28 mg/L PP-MPs	200 mg/L SP	0.14 mg/L PP-MPs + 200 mg/L SP	0.28 mg/L PP-MPs + 200 mg/L SP
Parameters	Mean ± SE(Min–max)	Mean ± SE(Min–max)	Mean ± SE(Min–max)	Mean ± SE(Min–max)	Mean ± SE(Min–max)	Mean ± SE(Min–max)
Exposure						
WBCs (Thousands/µL)	12.15 ± 0.23^ab^(11.15–12.7)	11.38 ± 0.24^bc^(10.69–12.1)	11.92 ± 0.28^ab^(11.1–12.7)	11.92 ± 0.28^ab^(11.1–12.7)	12.51 ± 0.26^a^ (11.76–13.31)	10.98 ± 0.154^c^(10.4–11.4)
Neutrophils (%)	11.11 ± 0.18^a^(10.78–12)	12.94 ± 0.36^bc^(11.88–14)	14.76 ± 0.43^d^(13.72–16)	11.65 ± 0.49^ab^(10.89–14)	14.23 ± 0.40^ cd^(13.07–15.4)	13.60 ± 0.44^ cd^(12.74–15)
Large lymphocytes (%)	58.37 ± 0.21^a^(57.83–59)	56.05 ± 0.57^c^(54.45–58)	52.24 ± 0.45^d^(50.97–54)	58.07 ± 0.45^a^(56–59)	58.07 ± 0.45^a^(56–59)	55.38 ± 0.51^c^(54–57)
Small lymphocytes (%)	25.20 ± 0.29^a^(24–26)	22.89 ± 0.37^b^(21.78–24)	19.57 ± 0.75^c^(16–21)	24.96 ± 0.34^a^(24–26)	25.17 ± 0.40^a^(23.96–26.4)	21.72 ± 0.16^b^(21–22)
Monocytes (%)	1.66 ± 0.33^a^(0.98–0.98)	2.55 ± 0.23^a^(2.18–3.3)	2.49 ± 0.23^a^(1.98–3)	1.82 ± 0.16^a^(1–2)	2.32 ± 0.21^a^(1.98–3)	2.49 ± 0.22^a^(1.96–3)
Eosinophils (%)	2.32 ± 0.21^a^ (1.96–3)	4.98 ± 0.73^abc^ (2.97–7)	4.49 ± 1.21^abc^ (1.96–8)	2.66 ± 0.33^ab^ (1.98–4)	5.47 ± 0.79^bc^ (3.27–7.7)	7.13 ± 0.31^c^ (6–8)
Lysozyme activity (LYZ) (mg/mL)	14.25 ± 0.25^a^ (14–150	10.5 ± 0.29^b^ (10–11)	8.5 ± 0.65^c^ (7–10)	14.25 ± 0.25^a^ (14–15)	13.25 ± 0.48^a^ (12–14)	13.25 ± 0.48^a^ (12–14)
Immunoglobulin (IgM) (µg/mL)	35 ± 0.41^a^ (34–36)	29.5 ± 0.65^c^ (28–31)	25 ± 0.41^d^ (24–26)	34.75 ± 0.25^a^ (34–35)	33 ± 0.41^b^ (32–34)	33.75 ± 0.25^ab^ (33–34)
Phagocytic activity (PA) (%)	24.5 ± 0.29^a^ (24–25)	19.75 ± 0.25^c^ (19–20)	16.25 ± 0.48^d^ (15–17)	24 ± 0.40^a^ (23–25)	21.5 ± 0.29^b^ (21–22)	22.5 ± 0.29^b^ (22–23)
Recovery						
WBCs (Thousands/µL)	12.02 ± 0.23^ab^ (11–12.6)	11.28 ± 0.24^bc^ (10.6–12)	11.12 ± 0.22^bc^ (10.6–12)	11.83 ± 0.26^ab^ (11–12.6)	12.38 ± 0.26^a^ (11.6–13.2)	10.88 ± 0.156^c^ (10.3–11.3)
Neutrophils (%)	11.02 ± 0.18^a^ (10.7–11.9)	12.82 ± 0.36b^c^ (11.8–13.9)	16.23 ± 0.48^d^ (15.1–17.6)	11.57 ± 0.50^ab^ (10.8–13.9)	14.08 ± 0.39^c^ (12.9–15.2)	13.48 ± 0.45^c^ (12.6–14.9)
Large lymphocytes (%)	57.77 ± 0.22^a^ (57.2–57.2)	55.48 ± 0.55^c^ (53.9–57.4)	57.47 ± 0.49^a^ (56.1–59.4)	57.57 ± 0.46^a^ (55.4–58.4)	61.05 ± 0.62^b^ (59.3–63.2)	54.85 ± 0.49^c^ (53.5–56.4)
Small lymphocytes (%)	24.97 ± 0.28^a^ (23.8–25.7)	22.67 ± 0.36^b^ (21.6–23.8)	21.53 ± 0.83^b^ (17.6–23.1)	24.77 ± 0.33^a^ (23.8–25.7)	24.92 ± 0.39^a^ (23.7–26.1)	21.52 ± 0.16^b^ (20.8–21.8)
Monocyte (%)	2.48 ± 0.23^a^ (1.9–3)	2.32 ± 0.20^a^ (2–3)	2.02 ± 0.18^a^ (1.1–2.2)	2.5 ± 0.22^a^ (2–3)	2.55 ± 0.22^a^ (2.2–3.3)	1.67 ± 0.33^a^ (1–3)
Eosinophils (%)	2.32 ± 0.22^a^ (1.9–3)	4.93 ± 0.72^abc^ (2.9–6.9)	4.95 ± 1.32^abc^ (2.2–8.8)	2.67 ± 0.33^ab^ (2–4)	5.4 ± 0.79^bc^ (3.2–7.6)	7.05 ± 0.31^c^ (5.9–7.9)
Lysozyme activity (LYZ) (mg/mL)	14.25 ± 0.25^a^ (14–15)	12.5 ± 0.29^a^ (12–13)	9.5 ± 0.65^b^ (8–11)	14.25 ± 0.25^a^ (14–15)	13 ± 0.41^a^ (12–14)	13.25 ± 0.48^a^ (12–14)
Immunoglobulin (IgM) (µg/mL)	34.75 ± 0.25^a^ (34–35)	35 ± 0.71^a^ (34–37)	28.75 ± 1.31^b^ (25–31)	34 ± 0.41^a^ (33–35)	32.75 ± 0.25^a^ (32–33)	33 ± 0.41^a^ (32–34)
Phagocytic activity (PA) (%)	24 ± 0.41^a^ (23–25)	23.5 ± 0.29^a^ (23–24)	18.5 ± 0.65^c^ (17–20)	23.5 ± 0.29^a^ (23–24)	21.5 ± 0.29^b^ (21–22)	21.5 ± 0.29^b^ (21–22)

Among the immunological parameters assessed, lysozyme (LYZ), immunoglobulin M (IgM), and phagocytic (PA) activity levels, there was a significant (*p* < 0.05) decrease when compared to the control. While the bioremediation with 200 mg/L SP in 0.14 and 0.28 mg/L PP-MPs exposed groups showed a significant (*p* < 0.05) decrease when compared to the control, there was a significant (*p* < 0.05) increase when compared to the same exposed groups without *Spirulina*. Following 45 days of recovery, similar trends of significant variations toward an increase or decrease were seen in comparison to the control group; however, in the 0.28 mg/L PP-MPs exposed group, there was a non-significant (*p* ≥ 0.05) increase in IgM (Table 1). Furthermore, the bioremediation with 200 mg/L SP in the 0.14 and 0.28 mg/L PP-MPs exposed groups following 45 days of recovery showed some variations in IgM and PA levels in comparison to the control group, while in comparison to the exposure period, the tested immunological parameters exhibited increase that was non-significant (*p* ≥ 0.05) in the PP-MP (0.14 mg/L) dose and significant (*p* < 0.05) in the 0.28 mg/L PP-MPs dose. The bioremediation with 200 mg/L SP in the 0.14 and 0.28 mg/L PP-MPs exposed groups following 45 days of recovery showed a non-significant (*p* ≥ 0.05) decrease in comparison to the exposure period.

### Antioxidant biomarkers

The antioxidants of *C. gariepinus* exposed to PP-MP (0.14 mg/L), PP-MP (0.28 mg/L), PP-MP (0.14 mg/L) + SP (200 mg/L), PP-MP (0.28 mg/L) + SP (200 mg/L), and SP (200 mg/L) as a positive control for 15 days and following 45 days of recovery are recorded in Table 2. With the exception of GST, all parameters SOD, CAT, and MAD indicated that there were significant differences (*p* < 0.05) between the control group and the treated groups. The parameters exhibited significance (SOD and CAT) or non-significance (GST) either declined with the increase of PP-MP doses from 0.0 inthe control to 0.28 mg/L (GST, SOD, and CAT) or increase with such a rise of doses (MAD) (Table 2). In comparison to the control group, all parameters showed non-significant (*p* > 0.05) changes in the 200 mg/L SP exposed group. Furthermore, the bioremediation with 200 mg/L SP in 0.28 mg/L PP-MPs showed significant (*p* < 0.05) increase in SOD in contrast to the identical exposed group that was devoid of *Spirulina*. The bioremediation with 200 mg/L SP in 0.14 and 0.28 mg/L PP-MPs exposed groups showed non-significant (*p* ≥ 0.05) rise in CAT in contrast to the identical exposed group that was devoid of *Spirulina*. The bioremediation with 200 mg/L SP revealed significant (*p* < 0.05) decrease in MAD in the 0.28 mg/L PP-MPs exposed group and non-significant (*p* ≥ 0.05) decrease in the 0.14 mg/L PP-MPs when compared to the same exposed groups without *Spirulina*. This suggests that *Spirulina* plays a bioremediation role in preventing PP-MP toxicity. Following 45 days of recovery, similar trends of significant variations toward a rise or decline were seen in all parameters in comparison to the control group; however, in the 0.14 and 0.28 mg/L PP-MPs exposed groups, there was a significant (*p* < 0.05) increase (Table 2). However, the antioxidant levels in the 0.14 and 0.28 mg/L PP-MPs exposed groups were significantly (*p* < 0.05) higher than throughout the exposure period.

**Table 2:** Antioxidant biomarkers (mean ± SE and min–max range) of the African catfish exposed to PP-MPs (0.14 mg/L), PP-MPs (0.28 mg/L), PP-MPs (0.14 mg/L) + SP (200 mg/L), PP-MPs (0.28 mg/L) + SP (200 mg/L), and SP (200 mg/L) as a positive control for 15 days followed by a 45-day period of recovery

Treatments	Control	0.14 mg/L PP-MPs	0.28 mg/L PP-MPs	200 mg/L SP	0.14 mg/L PP-MPs + 200 mg/L SP	0.28 mg/L PP-MPs + 00 mg/L SP
Parameters	Mean ± SE (Min–max)	Mean ± SE (Min–max)	Mean ± SE (Min–max)	Mean ± SE (Min–max)	Mean ± SE (Min–max)	Mean ± SE (Min–max)
Exposure						
Glutathione S-transferase (GST) (U/mL)	36 ± 1.08^a^ (34–39)	33.75 ± 1.11^a^ (31–36)	34.25 ± 0.48^a^ (33–35)	35.75 ± 1.25^a^ (33–39)	34 ± 0.91^a^ (32–36)	34 ± 0.91^a^ (32–36)
Superoxide dismutase (SOD) (U/mL)	3 ± 0^a^ (3–3)	2.25 ± 0.25^ab^ (2–3)	2 ± 0^b^ (2–2)	3 ± 0^a^ (3–3)	2.5 ± 0.29^ab^ (2–3)	2.5 ± 0.29^ab^ (2–3)
Catalase (CAT) (U/mL)	12.25 ± 0.75^a^ (11–14)	9.25 ± 0.25^b^ (9–10)	9 ± 0^b^ (9–9)	12.25 ± 0.75^a^ (11–14)	9.75 ± 0.48^b^ (9–11)	9.75 ± 0.48^b^ (9–11)
Malondialdhyde (MAD) (nmol/mL)	14.5 ± 0.64^a^ (13–16)	17.75 ± 0.25^b^ (17–18)	32.75 ± 0.25^c^ (32–33)	14.25 ± 0.63^a^ (13–16)	17.5 ± 0.29^b^ (17–18)	17.75 ± 0.25^b^ (17–18)
Recovery						
Glutathione S-transferase (GST) (U/mL)	35.75 ± 1.25^ab^ (33–39)	40.5 ± 1.32^b^ (37–43)	38.5 ± 2.18^ab^ (32–41)	35 ± 1.08^ab^ (33–38)	33 ± 0.91^a^ (31–35)	34 ± 0.91^a^ (32–36)
Superoxide dismutase (SOD) (U/mL)	3 ± 0^a^ (3–3)	3 ± 0^a^ (3–3)	2 ± 0^b^ (2–2)	3 ± 0^a^ (3–3)	2.25 ± 0.25^b^ (2–3)	2.5 ± 0.29^ab^ (2–3)
Catalase (CAT) (U/mL)	12.25 ± 0.75^a^ (11–14)	11.25 ± 0.25^ab^ (11–12)	10.25 ± 0.48^ab^ (9–11)	12 ± 0.71^ab^ (11–14)	9.75 ± 0.48^b^ (9–11)	9.75 ± 0.48^b^ (9–11)
Malondialdhyde (MAD) (nmol/mL)	14.25 ± 0.63^a^ (13–16)	21.25 ± 0.48^c^ (20–22)	37.5 ± 1.55^d^ (33–40)	14 ± 0.71^a^ (13–16)	17.5 ± 0.29^b^ (17–18)	17.5 ± 0.29^b^ (17–18)

At the 0.05 level of significance, values that share the same letters within a parameter do not differ (horizontal comparison)

The bioremediation with 200 mg/L SP in the 0.14 and 0.28 mg/L PP-MPs exposed groups following 45 days of recovery showed non-significance (*p* ≥ 0.05) in comparison to the exposure period. In these results, we noted that among the measured antioxidants, the malondialdehyde was significantly higher in all treated groups than in the control group over both times. However, it was highly significantly decreased in the bioremediation with 200 mg/L SP when compared to the same exposed groups without *Spirulina* in the recovery period.

### Effect of polypropylene on head kidney and the role of *Spirulina* using H&E and Sirius red staining

**Fig. 1:**
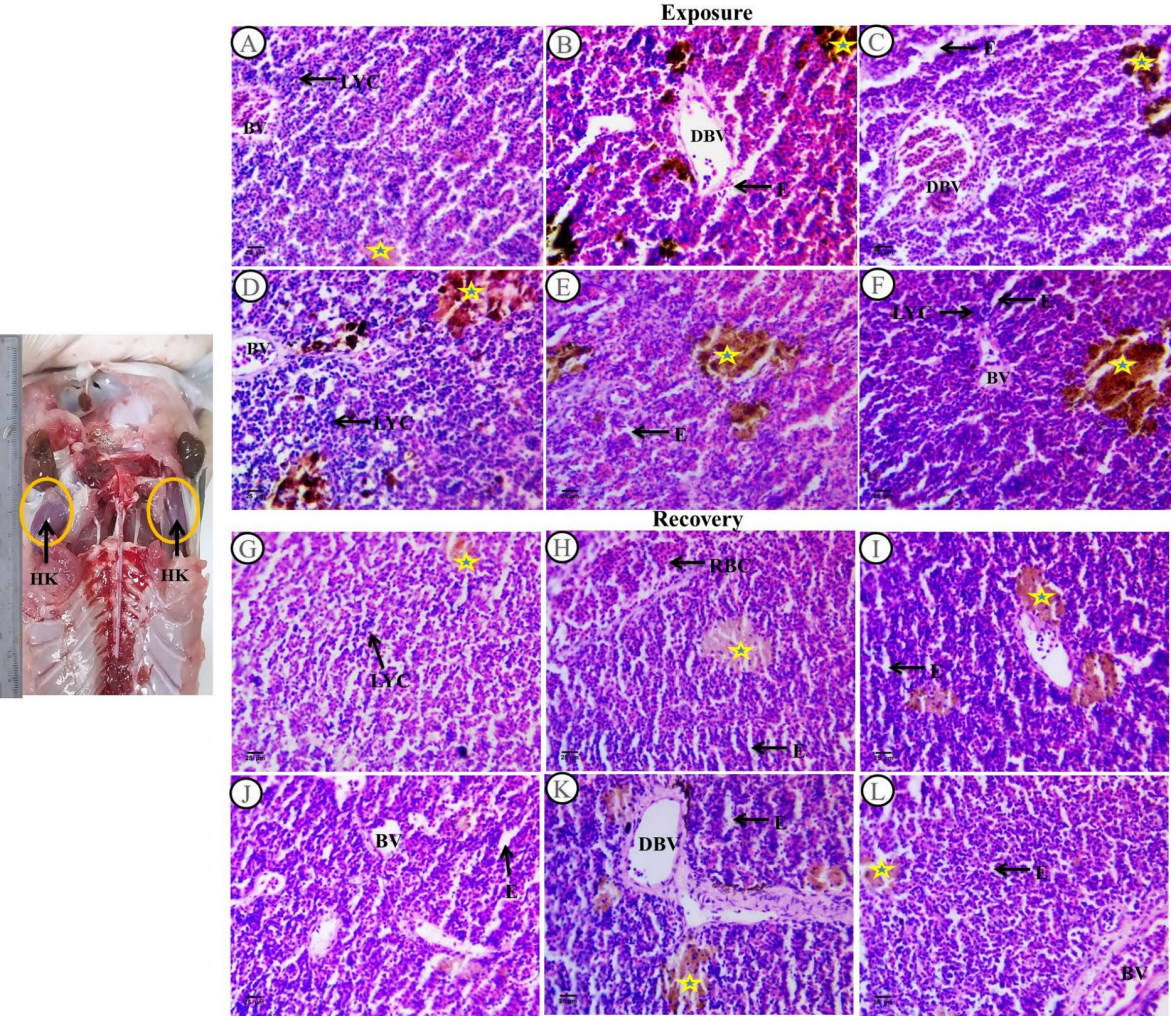
Sections of head kidney from control and treated fish stained with (H&E) (× 400) following an exposure period for 15 days and a recovery period for 45 days. **a** Dorsal view showing the active immune part of *C. gariepinus*, the head kidney that is formed by the two arms (HK). Sections** A**–**F** following a 15-day exposure period. **A** Section of the control group of fish showing the normal histological characteristics of *C. gariepinus*’s head kidney tissue. **B** Head kidney section from a fish treated with PP-MPs (0.28 mg/L) showing various degrees of damage. **C** Head kidney section from a fish treated with PP-MPs (0.14 mg/L). **D** Head kidney section from a fish exposed to *Spirulina* (200 mg/L). **E** Head kidney section from a fish treated with PP-MPs (0.28 mg/L) plus *Spirulina*. **F** Head kidney section from a fish treated with PP-MPs (0.14 mg/L) plus *Spirulina*. Sections **G**–**L** after a recovery period for 45 days. **G** Section from control fish group revealing typical histological characteristics of head kidney tissue of *C. gariepinus.* **H** Head kidney section from a fish treated with PP-MPs (0.28 mg/L). **I** Head kidney section from a fish treated with PP-MPs (0.14 mg/L). **J** Head kidney section from a fish treated with *Spirulina* (200 mg/L). **K** Head kidney section from a fish treated with PP-MPs (0.28 mg/L) plus *Spirulina*. **L** Head kidney section from a fish treated with PP-MPs (0.14 mg/L) plus *Spirulina*. Labeled structures are LYC, lymphocytes; BV, blood vessel; DBV, dilatation in blood vessel; E, edematous space; RBC, red blood cells; yellow star, melanomacrophage centers

Sections of *C. gariepinus* head kidney stained with H&E and treated with SP (200 mg/L) as a positive control, PP-MPs (0.28 mg/L), PP-MPs (0.28 mg/L) + SP (200 mg/L), PP-MPs (0.14 mg/L), and PP-MPs (0.14 mg/L) + SP (200 mg/L) for 15 days are shown in Fig. 1A–F. In catfish, the kidney is frequently a Y-shaped organ situated above the swim bladder along the body axis. The majority of the lower portion functions as a renal system and is a lengthy structure that is positioned parallel to the spinal column. The two arms combine to form the head kidney, the active immunological portion (Fig. 1a). The head kidney was covered by a thin capsule of connective tissue which lost renal tubules. However, it is composed of two distinct zones: a lymphoid zone that is highly stained and a non-lymphoid zone. The lymphoid zone was made up of clusters of tiny lymphoid cells that were grouped together around melanomacrophage centers and blood vessels. The non-lymphoid zone consists of hematopoietic system cells of several blood cell types.

In the control group, head kidney outer zone showed small patches of aggregated small lymphoid cells which contained deeply stained nuclei and red blood corpuscle dispersed in the interstitium of renal tissues. Small melanomacrophage centers contained yellow pigments (Fig. 1A). On the other hand, head kidney sections of fish treated with SP (200 mg/L) exhibited significantly better tissue structure. Head kidney outer zone was full of patches of aggregated small lymphoid cells which contained deeply stained nuclei and hemopoietic tissues mainly red blood corpuscle which were located at the boundary of blood vessels. Melanomacrophage centers contained different types of pigment coloration which were distributed between the interstitium of renal tissues in single or nodular shapes. There were few melanin cells full of dense melanin pigments and the main pigments were lipofusin as compared with the control one (Fig. 1D). Conversely, *C. gariepinus* sections exposed to dosages of 0.28 mg/L and 0.14 mg/L of PP-MPs showed various degrees of changes, including head kidney outer zone full of small patches of lymphocytes which was separated by edematous space and dilatation in blood vessel (contained red blood corpuscle). Melanomacrophage centers dispersed in the interstitium of renal tissues which contained black and brown pigments (Fig. 1B and C), while in *Spirulina* co-treatment (0.14 and 0.28 mg/L PP-MPs + 200 mg/L) exposed groups, there was little amelioration when compared with groups exposed to PP-MPs without *Spirulina*. Head kidney outer zone was full of increased large patches of lymphocytes which were separated by few edematous spaces. Melanomacrophage centers contained large yellow, few brown and black pigments that were dispersed in the interstitium of renal tissues. There were few blood corpuscles in blood vessel (Fig. 1E and F).

Following a 45-day recovery period, the head kidney tissue histological characteristics of the *C. gariepinus* were observed in the control group (Fig. 1G). When compared with the exposure period, head kidney sections from fish exposed to SP (200 mg/L SP) after the recovery period showed varying degrees of improvement. There were some histological changes such as head kidney outer zone showed dispersion of both lymphocytes and hematopoietic tissues mainly red blood corpuscles, angiogenesis in blood vessels which contains few blood corpuscles and decrease in melanomacrophage centers, which contained faint yellow pigments, and the edematous space in interstitium renal tissues also increased when compared with the exposure period (Fig. 1J). These results indicate the ameliorative role of *Spirulina* in the exposure period. The head kidney tissue in groups exposed to PP-MPs (0.28 mg/L and 0.14 mg/L) showed only slight improvement after the recovery period. Head kidney outer zone showed increased and dispersed small patches of lymphocytes and hematopoietic tissues mainly RBCs between interstitium of renal tissues. There were few shrunken edematous spaces and few melanomacrophage centers which contained yellow pigments in comparison with the same group in the exposure period. There were blood vessels containing few blood corpuscles near melanomacrophage centers (Fig. 1H and I), while in the *Spirulina* co-treatment (0.14 and 0.28 mg/L PP-MPs + 200 mg/L SP) exposed groups after the recovery period for 45 days, not much of an improvement was seen, compared with the same exposed groups. Head kidney outer zone showed increased nodules of lymphocytes which were separated by narrow edematous space. Hematopoietic tissues mainly red blood corpuscle dispersed in the interstitium renal tissues. There were melanomacrophage centers containing yellow pigments. Also, there was dilatation in blood vessel and thickening in connective tissues around it (Fig. 1K and L).

#### Collagen fibers examination

**Fig. 2:**
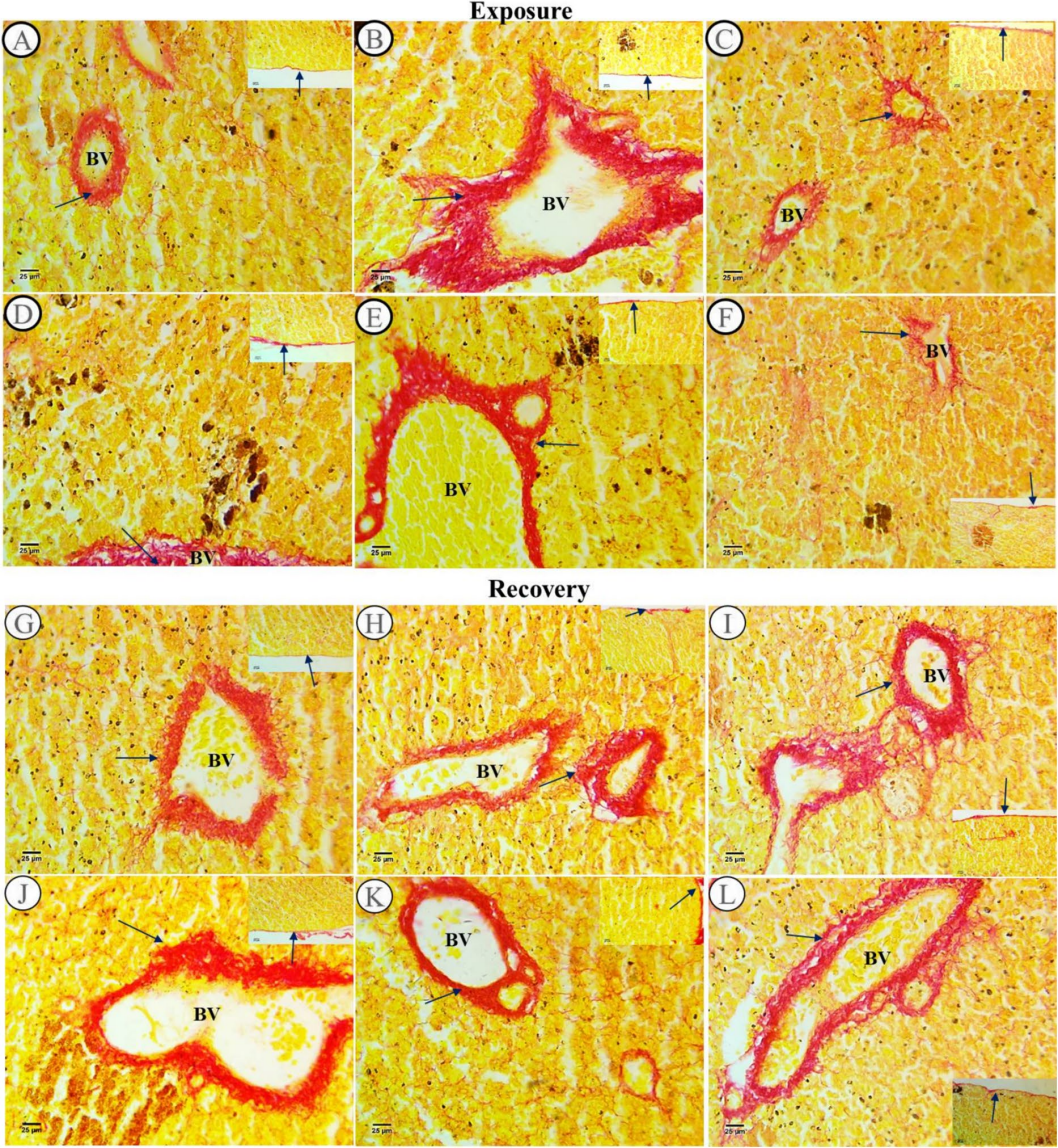
Picrosirius red-stained head kidney sections from control and exposed fish after an exposure period for 15 days and a recovery period for 45 days. **A** Section from a control fish displayed a typical distribution of collagen in the capsule and the wall of blood vessels (arrows). **B** and **C** Head kidney sections from a fish treated with 0.28 and 0.14 mg/L PP-MPs respectively. **D** Head kidney section from a fish treated with *Spirulina* (200 mg/L) showed an increase in collagenous fibers in both the capsule and the wall of blood vessels (arrows). **E **and** F** Head kidney sections from a fish treated with 0.28 and 0.14 mg/L PP-MPs plus *Spirulina* respectively showed a decrease in collagenous fibers in both the capsule and the wall of blood vessels. Sections** G**–**L** following a 45-day recuperation period. **G** Section from the control fish group showed a normal distribution of collagen in the capsule and the wall of blood vessels (arrows). **H** Head kidney section from a fish treated with PP-MPs (0.28 mg/L). **I** Head kidney section from a fish treated with PP-MPs (0.14 mg/L). **J** Head kidney section from a fish treated with *Spirulina* (200 mg/L). **K** head kidney section from a fish treated with PP-MPs (0.28 mg/L) plus *Spirulina*. **L** Head kidney section from a fish treated with PP-MPs (0.14 mg/L) plus *Spirulina* showed collagenous fibers in both the capsule and the wall of blood vessels (arrows). BV, blood vessel (× 400)

Collagenous fibers were detected in head kidney sections from all experimentally exposed groups using Sirius red stain. The collagen around the blood vessel wall and capsule was distributed normally in the control group (Fig. 2A), while the deposition of collagen fiber was increased in the SP (200 mg/L) exposed group in both locations (Fig. 2D). When comparing the PP-MPs (0.28 mg/L) exposed group to the control and SP (200 mg/L), the staining density around the blood vessels was higher and the capsule’s color was slightly faint (Fig. 2B). This intensity of color was declined in *Spirulina* co-treatment (0.28 mg/L PP-MPs + 200 mg/L SP) exposed group in both the capsule and the wall of blood vessels (Fig. 2E). At the time, in the PP-MPs (0.14 mg/L) and its *Spirulina* co-treatment (0.14 mg/L PP-MPs + 200 mg/L SP) exposed groups, the density of staining was decreased around blood vessels and capsule and more decrement was observed in PP-MPs (0.14 mg/L) (Fig. 2C) and its *Spirulina* co-treatment (0.14 mg/L PP-MPs + 200 mg/L SP) exposed groups (Fig. 2F) when compared with both PP-MPs (0.28 mg/L) and its *Spirulina* co-treatment exposed groups.

Following a 45-day recovery period, the control group’s collagen distribution in the capsule and blood vessel wall was normal (Fig. 2G). However, the SP (200 mg/L) exposed group displayed a little rise in the capsule and blood vessel wall, compared to the same exposed group (Fig. 2J). After the recovery time, the group treated with PP-MPs (0.28 mg/L) showed a little decrease compared to the same exposed group in the exposure period (Fig. 2H); however in its *Spirulina* co-treatment (0.28 mg/L PP-MPs + 200 mg/L SP), collagen fibers in both capsule and around blood vessels were nearly the same (Fig. 2K). At the time, the PP-MPs (0.14 mg/L) exposed group following the recuperation phase showed a little increment in both the capsule and around blood vessels when compared with the same group in the exposure treatment (Fig. 2I), while the PP-MPs (0.14 mg/L) with its *Spirulina* co-treatment exposed group following the recuperation phase revealed a rise in collagen contents around blood vessels when compared with the same group in the exposure treatment (Fig. 2L).

### Effect of polypropylene on spleen and the role of *Spirulina* using H&E and Sirius red staining

**Fig. 3:**
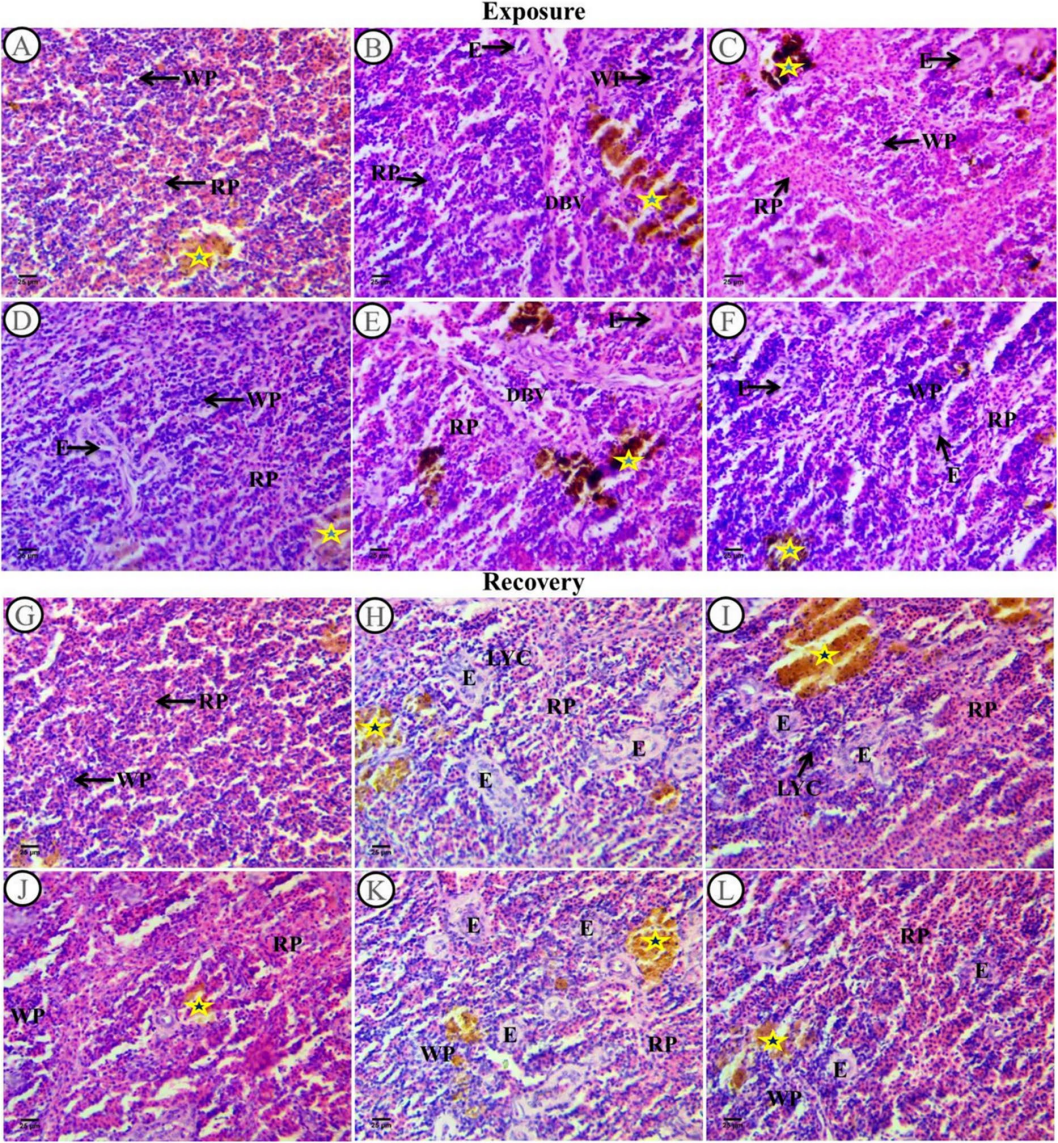
Spleen sections from control and treated fish stained with H&E (× 400) following a 15-day exposure period and a 45-day recovery period. Sections** A**–**F** after an exposure period for 15 days. **A** Section of the control fish group that displayed the typical histological characteristics of *C. gariepinus* spleen tissue. **B** Spleen section from a fish exposed to PP-MPs (0.28 mg/L) showing various degrees of damage. **C** Spleen section from a fish treated with PP-MPs (0.14 mg/L). **D** Head kidney section from a fish treated with *Spirulina* (200 mg/L). **E** Spleen section from a fish treated with PP-MPs (0.28 mg/L) plus *Spirulina*. **F** Spleen section from a fish treated with PP-MPs (0.14 mg/L) plus *Spirulina*. Sections **G**–**L** following a 45-day recuperation period. **G** Section from the control fish group that displayed the normal histological characteristics of *C. gariepinus* spleen tissue. **H** Spleen section from a fish treated with PP-MPs (0.28 mg/L). **I** Spleen section from a fish treated with PP-MPs (0.14 mg/L). **J** Spleen section from a fish treated with *Spirulina* (200 mg/L). **K** Spleen section from a fish treated with PP-MPs (0.28 mg/L) plus *Spirulina*. **L** Spleen section from a fish treated with PP-MPs (0.14 mg/L) plus *Spirulina*. Labeled structures are WP, white pulp; RP, red pulp; DBV, dilatation in blood vessel; E, ellipsoid bodies; LYC, lymphocytes; yellow star, melanomacrophage centers

Sections of the spleen of catfish treated with SP (200 mg/L) as a positive control, PP-MPs (0.28 mg/L), PP-MPs (0.28 mg/L) + SP (200 mg/L), PP-MPs (0.14 mg/L), and PP-MPs (0.14 mg/L) + SP (200 mg/L) for 15 days are displayed in Fig. 3A–F. The control group exhibited the three main splenic structures: red pulp, sinusoid capillaries (open capillaries), and the interconnecting system of splenic cords. However, *C. gariepinus* had poorly developed white pulp, which is mainly composed of widely distributed lymphoid cells that form a marginal zone. Melanomacrophage cells (MMCs) and clusters of blood sinuses are found in the white and red pulps, together with ellipsoid structures (Fig. 3A). Fish spleen sections treated with SP (200 mg/L) revealed amelioration in splenic morphology; the red and white pulps were clearly visible, along with the presence of melanomacrophage, a rise in ellipsoid bodies found in the white pulp’s core, and an increase in lymphocytes aggregating in the white pulp, while red pulp is full of hematobiotic tissues especially red blood corpuscle (Fig. 3D). Conversely, *C. gariepinus* sections exposed to PP-MPs (0.28 mg/L) displayed a range of alterations; among them, the white pulp increased in size containing newly formed lymphocytes, vascular dilatation of a blood vessel containing blood corpuscles, and indistinct boundaries separating the red and white pulps. Additionally, the melanomacrophage centers expanded and contained hemosiderin, which is a big, granular, irregular brown pigment. In both the white and red pulps, the ellipsoid structures showed up as terminal capillaries encased in a sheath of acidophilic fibrous connective tissue and scattered lymphocytes. The lining of these structures showed deformations. The red pulp was shrunken and contained dispersed hematopoietic tissues (Fig. 3B). In sections from *C. gariepinus* exposed to PP-MPs (0.14 mg/L), the red pulp grew larger and contained more hematopoietic tissues, primarily red blood cells. The white and red pulps boundaries were markedly evident in comparison with the PP-MPs (0.28 mg/L) exposed group. There was shrinkage in the white pulp with a small number of freshly generated lymphocytes. The presence of big, irregular, black and brown granular pigments (melanin and hemosiderin) in melanomacrophage centers was observed. Ellipsoid structures were observed in both the white and red pulps showing deformation in their lining and thickening of their sheath (Fig. 3C). In the *Spirulina* co-treatment (0.14 and 0.28 mg/L PP-MPs + 200 mg/L SP) exposed groups, marked improvements were observed in these groups when compared to the PP-MPs exposed groups without *Spirulina*. There was a rise in the foci of newly formed lymphocytes that observed in the white pulp. The melanomacrophage centers were noticed with large, irregular, black and brown granular pigments (melanin & hemosiderin) (Fig. 3E and F). However, still there were some signs of pathological alterations such as white and red boundaries were dispersed and not evident, dilation of the blood vessels filled with blood corpuscle, widening of ellipsoid structures, and deformation in their lining which encircled by a robust sheath of acidophilic fibrous connective tissue accompanied by dispersed lymphocytes. However, these signs of splenic structure changes were in a little degree in 0.14 mg/L PP-MPs + 200 mg/L SP (Fig. 3F).

After a 45-day recuperation period, the control group showed typical *C. gariepinus* splenic architecture (Fig. 3G). Splenic morphology of fish treated with SP (200 mg/L SP) after the recuperation period showed some pathological alterations when compared with the same group in the exposure period; this indicated the ameliorative role of *Spirulina* in the exposure period. The white pulp contained decreased foci of lymphocytes. Both white and red pulps showed ellipsoid structures, and their linings were deformed, while red pulp was full of hematopoietic tissues especially huge numbers of red blood corpuscle. There was an increase in the ellipsoid bodies and the presence of yellow pigments in melanomacrophage centers (Fig. 3J). When compared to the same exposed groups in the exposure period, the PP-MPs (0.14 and 0.28 mg/L) exposed groups after the recovery period displayed a noticeable improvement in splenic architecture such as increase in ellipsoid structures with great amelioration in their lining (cubic epithelium) and their sheath which was surrounded by acidophilic fibrous connective tissues and dispersed lymphocytes (Fig. 3H and I). There were melanomacrophage centers with big, irregular, and yellow granular pigments (lipofuscin). However, white and red pulp boundaries were not clear obviously and the white pulp contained few aggregated foci of newly formed lymphocytes. In the *Spirulina* co-treatment (0.14 and 0.28 mg/L PP-MPs + 200 mg/L SP) exposed groups after the recovery period for 45 days, marked improvement was noticed in these groups when compared with the same groups without *Spirulina*, but minimal improvement was observed in comparison to the same exposed groups (Fig. 3K and L). There were dispersed or foci of newly formed lymphocytes in the white pulp and an increase in melanomacrophage centers with large, irregular, yellow, and granular pigments (lipofuscin). The boundary separating the red and white pulps was unclear. There was an increase in ellipsoid structures with great amelioration in their lining (cubic epithelium), and their sheath was thick.

#### Collagen fibers examination

**Fig. 4:**
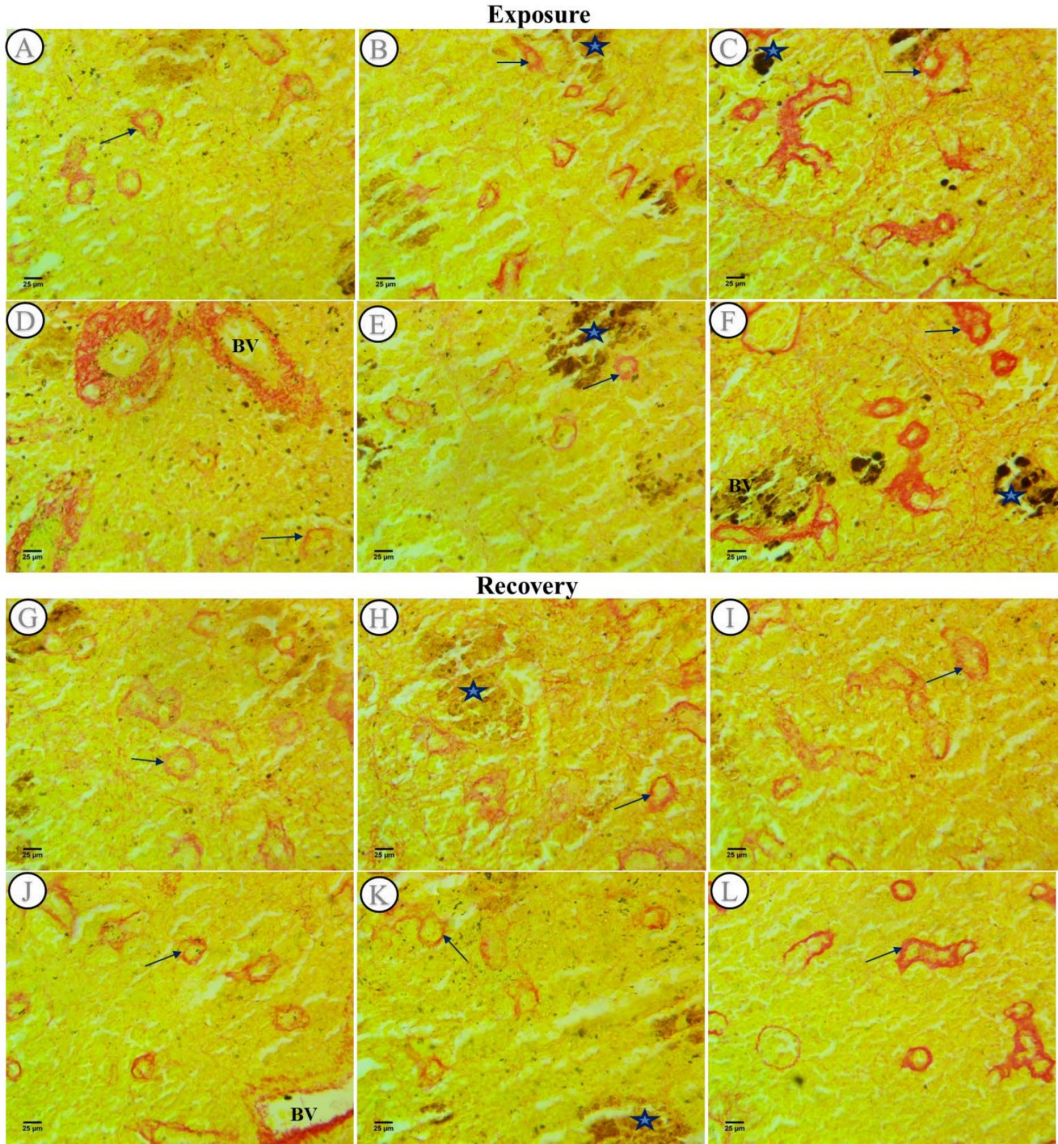
Picrosirius red-stained spleen sections from control and treated fish after an exposure period for 15 days and a recovery period for 45 days. **A** Section from a control fish displayed a normal distribution of collagen in the connective sheath of ellipsoid bodies (arrows). **B** and **C** Spleen sections from a fish treated with 0.28 and 0.14 mg/L PP-MPs, respectively, showed a rise in collagenous fibers in the connective sheath of ellipsoid bodies. **D** Spleen section from a fish treated with *Spirulina* (200 mg/L) showed an increase in collagenous fibers in the connective sheath of ellipsoid bodies (arrows). **E **and** F** Spleen sections from a fish treated with 0.28 and 0.14 mg/L PP-MPs plus *Spirulina*, respectively. Sections** G**–**L** after a recovery period for 45 days. **G** Section from the control fish group displayed a typical distribution of collagen in the connective sheath of ellipsoid bodies (arrows). **H** Spleen section from a fish treated with PP-MPs (0.28 mg/L). **I** Spleen section from a fish treated with PP-MPs (0.14 mg/l). **J** Spleen section from a fish treated with *Spirulina* (200 mg/L). **K** Spleen section from a fish treated with PP-MPs (0.28 mg/L) plus *Spirulina*. **L** Head kidney section from a fish treated with PP-MPs (0.14 mg/L) plus *Spirulina* displayed collagen in the connective sheath of ellipsoid bodies (arrows). Blue star, melanomacrophage (× 400)

Collagenous fibers were detected in the spleen sections of every experimentally exposed group using Sirius red stain: in the control group, the collagen distribution around the ellipsoid bodies’ connective sheath was normal (Fig. 4A), while in the SP (200 mg/L) group, an increase in staining of collagen in the connective sheath of ellipsoid bodies was revealed when compared to the control group (Fig. 4D). In the PP-MPs (0.28 mg/L) exposed group, there was a rise in the intensity of staining and the numbers of ellipsoid bodies (Fig. 4B) as compared with the collagen in the same group with *Spirulina* (0.28 mg/L PP-MPs + 200 mg/L SP) which showed a decrease in both intensity and the numbers of these bodies (Fig. 4E). At the time, the PP-MPs (0.14 mg/L) and its *Spirulina* co-treatment (0.14 mg/L PP-MPs + 200 mg/L SP) exposed groups (Fig. 4C and F) showed amplification in collagenous fiber intensity and numbers but the maximum rise was observed in the PP-MPs (0.14 mg/L) exposed group (Fig. 4C) as compared with all exposed groups. It was observed that the melanomacrophage centers contained few dark pigments in both the PP-MPs (0.28 mg/L) and its *Spirulina* co-treatment exposed groups and these pigments were increased in the PP-MPs (0.14 mg/L) and its *Spirulina* co-treatment exposed groups.

Following a 45-day recovery period, the control group’s collagen distribution in the ellipsoid bodies’ connective sheath was normal (Fig. 4G), while SP (200 mg/L) group showed shrinkage in the size of ellipsoid bodies and slight increase in collagen staining when compared with the control group (Fig. 4J). In PP-MPs (0.28 mg/L) exposed group after the recovery period, the intensity of staining and the numbers of ellipsoid bodies were more or less similar to the control group (Fig. 4H), while in its *Spirulina* co-treatment exposed group, there was marked decrease in the intensity of collagen fibers (Fig. 4K). At the time, the PP-MPs (0.14 mg/L) and its *Spirulina* co-treatment treated groups following the recuperation period showed amplification in collagenous fiber intensity but a maximum rise was observed in its *Spirulina* co-treatment exposed group as compared with all groups (Fig. 4I and L).

### Measurements of melanomacrophage centers in the head kidney and spleen

*Clarias gariepinus*’s melanomacrophage centers in the head kidney and spleen after 15 days of exposure and 45 days of recuperation are provided in Table 3. The number and size of melanomacrophage centers in the head kidney exhibited a significant increase with the rise of PP-MP dosages ranged from 0.28 mg/L to 0.0 in the control. In the 200 mg/L SP treated group, there was a highly significant rise in melanomacrophage size and number in comparison to the control group. Furthermore, the bioremediation with 200 mg/L SP in the 0.14 and 0.28 mg/L PP-MPs treated groups showed a non-significant decrease in melanomacrophage number and a significant reduction in their size when compared to the same exposed groups without *Spirulina*. After the 45-day recovery, all treated groups showed a non-significant (*p* ≥ 0.05) reduction in melanomacrophage number in contrast to the exposure period (Table 3), while there were non-significant (*p* ≥ 0.05) variations in melanomacrophage size in all treated groups compared to the control and the exposure period. In the spleen, the adverse effects of the pollutant were also recorded on melanomacrophage size and number. Similar trends of significant changes in the direction of a rise or fall were seen in melanomacrophage size and number of the spleen as in the head kidney (Table 3).

**Table 3:** Variations in melanomacrophage center (MMCs/µm^2^) size and number in head kidney and spleen of the African catfish exposed to PP-MPs (0.14 mg/L), PP-MPs (0.28 mg/L), PP-MPs (0.14 mg/L) + *Spirulina* (200 mg/L), PP-MPs (0.28 mg/L) + *Spirulina* (200 mg/L), and *Spirulina* (200 mg/L) as a positive control for 15 days followed by a 45-day period of recovery (*N* = 3 per each replicate)

Treatments	Size of melanomacrophage µm^2^		Number of melanomacrophage	
	Exposure period	Recovery period	Exposure period	Recovery period
	Mean ± SE (min–max)	Mean ± SE (min–max)	Mean ± SE (min–max)	Mean ± SE (min–max)
Head kidney				
Control	29,989 ± 10,146.3^a^ (0–98,984.4)	29,981 ± 10,146.3^a^ (0–98,984.4)	0.93 ± 0.25^a^ (0–3)	0.87 ± 0.16^a^ (0–2)
PP-MPs (0.28 mg/L)	44,840 ± 6720.4^b^ (0–116,000)	31,980 ± 9424.3^a^ (0–91,128.1)	2.33 ± 0.27^c^ (1–4)	0.87 ± 0.22^a^ (0–3)
PP-MPs (0.14 mg/L)	42,265 ± 6819.2^c^ (2734.4–100,000)	42,817 ± 4527.8^a^ (12,265–87,268)	1.53 ± 0.24^abc^ (0–3)	1.26 ± 0.27^a^ (0–3)
***Spirulina*** (SP)	58,363 ± 9501.7^b^ (25,965.6–170,000)	54,480 ± 17,225^a^ (0–280,000)	3.27 ± 0.28^d^ (2–5)	1.53 ± 0.13^a^ (1–2)
PP-MPs (0.28 mg/L) + SP	25,948 ± 3679.4^c^ (0–62,615.6)	41,915 ± 9833.3^a^ (0–138,000)	2.13 ± 0.21^bc^ (1–4)	1.73 ± 0.28^a^ (0–4)
PP-MPs (0.14 mg/L) + SP	112,620 ± 23,352.8^b^ (0–224,000)	31,255 ± 6890^a^ (0–91,615.6)	1.33 ± 0.21^ab^ (0–3)	1.27 ± 0.23^a^ (0–3)
Spleen				
Control	36,852 ± 9106.7^a^ (0–101,000)	55,914 ± 15,521.9^a^ (0–152,000)	1.67 ± 0.25^a^ (0–4)	1.73 ± 0.21^a^ (0–3)
PP-MPs (0.28 mg/L)	75,295 ± 14,031.9^b^ (15,796.9–161,000)	36,992 ± 7985.5^a^ (0–122,000)	2.8 ± 0.2^c^ (2–4)	2.26 ± 0.37^a^ (0–5)
PP-MPs (0.14 mg/L)	53,852 ± 4858.2^c^ (0–69,581.2)	31,192 ± 11,585.7^a^ (0–174,000)	2.67 ± 0.23^c^ (1–4)	1.87 ± 0.25^a^ (0–3)
***Spirulina*** (SP)	36,701 ± 10,822.3^a^ (0–80,871.9)	45,917 ± 11,164.2^a^ (0–155,000)	0.8 ± 0.2^b^ (0–2)	1.93 ± 0.31^a^ (0–4)
PP-MPs (0.28 mg/L) + SP	40,111 ± 7778.4^d^ (8050–143,000)	55,874 ± 12,834^b^ (20,450–178,000)	2.6 ± 0.23^c^ (1–4)	2 ± 0.27^a^ (0–4)
PP-MPs (0.14 mg/L) + SP	37,359 ± 7684.5^c^ (5696.9–132,000)	43,897 ± 8560.7^a^ (16,512.5–130,000)	2.8 ± 0.29^c^ (0–4)	1.73 ± 0.24^a^ (0–4)

Different letters in the same column indicate significant difference at *p* < 0.05

## Discussion

One of the most important issues confronting the globe today is environmental toxicant pollution. Because these pollutants wind up in the aquatic surroundings wherever they occur, fish are especially exposed to them (Fırat et al. 2011; Kolawole and Iyiola 2023). One of these pollutants is PP-MPs emitted from a variety of sources, especially paper cups which are often used in daily life, ending up in the environment and continuing to exist in environments.

Blood serves as a patho-physiological gauge of body health and the hematological parameters reveal any abnormality in response to environmental stress (Anderson and Siwicki 1995; Sandnes et al. 1988). White blood cells (WBCs) play a vital role in the innate immune system and help regulate immune functions within an organism. Because of this, they are commonly used as indicators of fish health (Fazio 2019). The marked decrease in WBC counts observed in the groups exposed to PP-MPs suggests that these particles may have an immunosuppressive effect. Since WBCs are essential for defending against infections, their decline could increase the fish’s vulnerability to disease (Fazio 2019; Magnadottir 2010; Shirmohammadi et al. 2025).

Neutrophils are important for the immune responses development, because they activate a non-specific immunity mechanism in fish (Ainsworth 1992; Mokhtar et al. 2023). In this investigation, leucocyte levels changed with increasing PP-MPs doses: WBCs, large and small lymphocytes, and monocytes decreased, while neutrophils and eosinophils increased. This finding aligns with earlier studies on acute blood-related effects in fish exposed to toxic substances, particularly in *Sarotherodon melanotheron* (Akinrotimi et al. 2011). An increase in neutrophils indicates an immune response to the toxicants. Studies have demonstrated that changes in fish hematological indices caused by toxicants depend on several factors, including the species, type of chemical, and duration of exposure (Shaalan and Sayed 2025). These changes are also influenced by the chemical composition, quality, and concentration in the water (Shaalan and Sayed 2025). Such exposure can lead to both reversible and irreversible alterations in fish blood parameters (Akinrotimi 2010). According to Xu Chang et al. (2018), the number of neutrophils in the Nile tilapia’s head kidney in brackish water was noticeably higher in contrast to that in freshwater. It is known that differences in leukocyte counts can be a sensitive indicator of environmental stress that is sensitive to changes (Li et al. 2011).

According to Singh and Tandon (2009), and Miteva et al. (2024), lymphocytes are in charge of the immune response because they produce chemicals and antibodies that serve as an anti-infection barrier (Musa et al. 2013). As circulating lymphocytes are destroyed, lymphoid organs may respond by raising the blood’s lymphocyte count (Shah and Altindağ 2005; Shahjahan et al. 2022). Lymphocyte levels were significantly reduced in the PP-MPs exposed groups compared to the control, possibly indicating lower sensitivity to the treatment or greater variability in their response. Likewise, monocyte counts also decreased across all treated groups, suggesting a general suppressive effect on this leukocyte type. These findings are consistent with observations by Shaalan and Sayed (2025) in *Cyprinus carpio* (common carp) exposed to pharmaceutical micropollutants. Stress can also result in changes in the amount of leukocytes, which might affect the fish’s blood physiology and weaken its immune system, leaving it more susceptible to sickness (Ariweriokuma et al. 2016).

A number of negative responses, including gastrointestinal tract blockage, delayed development, oxidative damage, cellular lesions, and problems with metabolism, have been linked to MP consumption, according to numerous scientific investigations (Cole et al. 2011; Ding et al. 2018; Eerkes-Medrano et al. 2015). According to El Euony et al. (2020), numerous substances have the capacity to be immunotoxic, which is frequently manifested as inflammation in healthy animals and worse disease. Fish’s ability to defend against infection can be disrupted and influenced by environmental pollutants, which can affect the fish’s innate immune response (Datta et al. 2009; Debbarma et al. 2024). Numerous extensively documented indicators for immunological dysfunction have been employed in investigations on fish immunotoxicity. The innate immune system’s defense against pathogenic changes, such as lysozymes, is largely dependent on macrophages (Brott and Clarke 2019; Sayed et al. 2022). According to reports, the production of immunological enzymes was hampered by the decline in hemocyte counts (Hong et al. 2020). In this investigation, there was a significant (*p* < 0.05) reduction in the immunological parameters, LYZ, IgM, and PA activity levels in contrast to the control group, while the bioremediation with 200 mg/L SP in 0.14 and 0.28 mg/L PP-MPs treated groups showed significant (*p* < 0.05) rise when compared to the same exposed groups that did not receive *Spirulina*. El Euony et al. (2020) reported that investigations on *C. gariepinus* also demonstrated immunotoxic effects, especially decreased serum lysozyme activity and phagocytic activity after exposure to thiamethoxam. Similar results are found in other investigations that follow pesticide exposure to Voliam flexi® in catfish (Mohamed et al. 2022) and waterborne pyrogallol exposure in African catfish (Hamed et al. 2024). Furthermore, microplastics may have detrimental effects on *C. gariepinus* immunological factors (Li et al. 2021b); additionally, deregulation of transcripts may function to prevent cells from growing and proliferating in Takifugu rubripes, the tiger pufferfish, in response to hypoxia (Shang et al. 2022). Fish and other animals have a natural immune response that involves lysozymes as a key defense molecule against foreign invaders and oxidative damage prevention (Guha et al. 2025; Saurabh and Sahoo 2008). Strong drops in the LYZ level are typically the result of a decrease in phagocytic activity (Khalil et al. 2020). Comparably, it was revealed that treating *C. carpio* with chlorpyrifos and cypermethrin (Soltanian and Fereidouni 2017) and treating *rainbow trout* and *gilthead seabream* with deltamethrin (Guardiola et al. 2014) resulted in much lower levels of lysozyme and IgM. IgM is one of the ubiquitous and significant indicators of humoral immunity in animals (Saurabh and Sahoo 2008). Common carp treated with diazinon and bifenthrin had lower plasma IgM values than fish that were not treated; this reduction may have been brought about by inhibiting the growth of hepatocytes and lymphocytes (natural killer cells, T cells, and B cells), which are the main sources of humoral immunity by producing antibodies and other immune proteins (Banaee 2013).

Exposure to MPs can lead to an increase in ROS within fish due to their ability to penetrate cells, where they either consume existing antioxidants or directly stimulate ROS production through their toxic effects (Jo et al. 2025; Shirmohammadi et al. 2024). Fish possess a well-developed antioxidant defense system, and changes in this system are key indicators of oxidative stress resulting from MP exposure (Pei et al. 2022). The oxidative stress index (OSI) serves as a comparative measure reflecting the balance between free radical generation and the antioxidant response (Sayed and Khalil 2016). Antioxidant enzymes like GST, SOD, and CATcan scavenge excess ROSto shield the body tissues from the damaging effects of oxidative stress (Dawood et al. 2020; El Basuini et al. 2020; Moustafa et al. 2020). Superoxide dismutase is the primary enzyme involved in mitigating oxidative stress in fish, facilitating the conversion of superoxide radicals into hydrogen peroxide (Jo et al. 2025). This enzyme exists in three forms: Cu/Zn-SOD, Mn-SOD, and Fe-SOD depending on the metal ion present at the active site. Cu/Zn-SOD is commonly found in both intracellular and extracellular spaces of prokaryotic and eukaryotic organisms and plays a crucial role in defending tissues against ROS-induced damage (Shi et al. 2022). Alongside SOD, CAT acts as a major antioxidant enzyme that decomposes hydrogen peroxide into water and oxygen. CAT exists in three variants: monofunctional catalases, bifunctional catalase-peroxidases, and manganese catalases (Shen et al. 2020; Shiry et al. 2023). In this investigation, fish exposed to PP-MP displayed a decrease in GST, SOD, and CATwith the rise of PP-MP concentrations from 0.0 inthe control to 0.28 mg/L. Consistent with our findings, SOD activity was reduced in zebrafish subjected to combined cadmium and MP exposure (Lu et al. 2016). Moreover, CAT activity was also diminished in zebrafish exposed to polystyrene microplastics (Wan et al. 2019), which has been attributed to the energy demands of managing oxidative stress triggered by microplastic exposure (Hamed et al. 2020). Contrary to the current findings, several studies have reported increased activities of antioxidant enzymes following microplastic exposure. Romano et al. (2020) observed elevated SOD and CAT levels in goldfish (*Carassius auratus*) exposed to PVC, suggesting a protective antioxidant response. Similarly, Ding et al. (2018) and Liu et al. (2024) reported increased SOD activity in Nile tilapia (*O. niloticus*) and grass carp (*Ctenopharyngodon idellus*), respectively, indicating activation of antioxidant defenses against MP-induced oxidative stress. Zitouni et al. (2021) also found enhanced SOD and CAT activities in European seabass (*Dicentrarchus labrax*), further supporting the notion that MP exposure can stimulate antioxidant mechanisms to counteract ROS.

The bioremediation with 200 mg/L SP significantly increased the activities of GST and CAT and reduced MAD when compared to the same exposed groups without *Spirulina*. This confirms the bioremediative role of *Spirulina* against the toxicity of PP-MPs. Malondialdehyde can harm a cell’s DNA, proteins, and cytoplasm since it is a consequence of elevated ROS levels and lipid peroxides (Yao et al. 2010). Variations in MDA levels in biological organisms are widely recognized as markers of oxidative stress and cellular damage (Henning and Weber 2021). In these results, we noted that, among the measured antioxidants, malondialdehyde was highly significantly raised in comparison to the control group in both periods in all exposed groups, confirming that PP-MPs produced ROS levels that caused oxidative stress. Prolonged or severe oxidative stress can result in chronic inflammation, tissue damage, functional impairments (Bobori et al. 2022), and even mortality (Xu et al. 2023). Furthermore, it may weaken the immune system, heightening vulnerability to infections and immune-related disorders (Sendra et al. 2021). Wang et al. (2024) indicate that waterborne MPs significantly raise MDA levels compared to MPs ingested through food. This implies that MPs in water present a higher oxidative risk, likely due to their more direct interaction with fish tissues (Bevan et al. 2020; Yu et al. 2022).

In fish, histopathological biomarkers have been mainly employed to determine and assess the harmful consequences of exposure to pollutants (Ribeiro et al. 2006). Fish immune function may be negatively impacted by such histopathological alterations, which could be the consequence of the spleen’s incapacity to recognize and remove PP-MPs as an alien xenobiotic substance. These results align with those of recent research by Sun et al. (2019), who documented inflammatory reactions, antioxidative stress, and splenic lesions in catfish (*Pelteobagrus fulvidraco*) as a consequence of cadmium accumulation, Hamed et al. (2024) as a result of waterborne pyrogallol exposure in African catfish and Yan et al. (2024) as a result of PP-MPs exposure. The results of this investigation, along with those of other studies, showed that the frequency of histopathological damage in the main organs’ tissues, especially the head kidney and spleen, was positively correlated with the immune response (e.g., the toxic effect of PP-MPs discharged from several origins, principally paper cups, on numerous immunological components). The spleen is an essential organ for hematopoiesis and immunity; in our investigation, the spleen’s histology after exposure to PP-MPs doses (0.28 mg/L and 0.14 mg/L) demonstrated variable degrees of toxicity, including white pulp increased in size containing newly formed lymphocyte, vascular dilatation of blood vessel, and the white and red pulp boundary that was unclear. Moreover, the melanomacrophage centers expanded with large, irregular, brown, and granular pigments (hemosiderin). The ellipsoid structures’ lining was deformed, and the red pulp had shrunk. As the concentration of PP-MPs increased, these signs of tissue damage became more severe as the damage seemed to be dose-dependent. According to Kranz and Gercken (1987), Mohamed et al. (2022), and Hamed et al. (2024), this might be the outcome of lymphocyte aggregation as an immunological reaction.

The spleen is the only organ known to have an abundance of lymphocytes and macrophages among the lymph nodes in bony fish (Nemcsók et al. 1984). According to Xia and Triffitt (2006), macrophages have the ability to absorb a variety of heterogeneous particles, including macromolecules, bacteria, and affected or apoptotic tissues. Additionally, they can restore tissue integrity by triggering a protective inflammatory response and producing effector molecules (Atri et al. 2018). In the present investigation, the number and size of melanomacrophage centers in the head kidney and spleen exhibited a significant rise when PP-MP dosages were increased from 0.0 mg/L in the control to 0.28 mg/L. Consequently, the immunotoxic potential of PP-MPs was evident from the increase in the melanomacrophage population in exposed fish spleen and head kidney. Along with this study, Yan et al. (2024) demonstrated that the MMCs in fish spleen exposed to PP-MPs were larger, more numerous, and more colorful than those of fish that were not exposed. As demonstrated by our findings, the spleen and head kidney both experienced macrophage aggregation. This indicated that PP-MPs caused these immune organs to experience increased oxidative stress and changed their immunological status by producing pro-inflammatory chemokines and cytokines that allowed phagocytosis to identify and remove foreign antigens as reported by Liu et al. (2023) after the exposure of *Cyprinus carpio* var. larvae to PVC microplastics.

Since the head kidney is the primary hemopoietic tissue in fish, HKM have been employed to study non-specific immunological activation (Romeo et al. 2000). In our investigation, the head kidney’s histology following exposure to PP-MPs doses (0.28 mg/L and 0.14 mg/L) demonstrated various degrees of changes, including head kidney outer zone full of small patches of lymphocytes which were separated by edematous space and dilatation in blood vessel. Melanomacrophage centers dispersed in the interstitium of renal tissues which contained black and brown pigments. Similarly, Datta et al. (2009) confirmed that chronic exposure to micromolar amounts of arsenic was found to have an impact on the histoarchitecture of HK.

In this study, the majority of histological and histochemical markers in *Spirulina* co-treatment (0.14 and 0.28 mg/L PP-MPs + 200 mg/L SP) exposed groups were markedly improved when compared to PP-MPs exposed groups without *Spirulina*. This was consistent with other researches, Sayed et al. (2023), Sayed et al. (2015), and Abdullah et al. (2024) with different pollutants. In accordance with Jeyavani et al. (2023) and Eid et al. (2024), the current investigation showed that *C. gariepinus* exposed to PP-MPs for up to 15 days generated reactive oxygen species (ROS) levels that resulted in oxidative stress, which damages the organs’ tissues. The harmful effects on the spleen and head kidney function were mitigated by co-treatment with *Spirulina* maybe because of its ability to scavenge and decrease ROS (Abdel-Daim et al. 2020; Salah El-Din et al. 2021; Sayed et al. 2023). The rich composition of *Spirulina*, including β-carotene, C-phycocyanins, minerals, vitamins, proteins, carbs, and lipids, contributes to its functioning and demonstrates its antioxidative activity (Abdel-Daim et al. 2020; Upasani and Balaraman 2003).

It was evident that there had been a noticeable improvement in leucocytes, immunological parameters, and antioxidant biomarkers (GST, SOD, and CAT) following 45 days of recuperation under normal conditions. While MAD did not recover in all PP-MPs exposed groups, the head kidney and spleen tissues exhibited little amelioration, indicating that the morphological restoration of immunological tissues took longer. As mentioned by Ribeiro et al. (2017) and Hamed et al. (2020), there are two possible explanations for this: either animal tissues are incapable of removing microplastics, or they can regenerate after a recovery period. Furthermore, Paul-Pont et al. (2016) reported that certain antioxidant enzymes continued to substantially highly indicate the requirement for a stronger capacity to neutralize free radicals even after the elimination period. This highlighted the risk posed by PP-MPs discharged from a variety of origins, especially paper cups that are used extensively in daily life and usually wind up in the environment as reported in our previous study (Eid et al. 2024).

## Conclusion

This study demonstrates that short-term exposure (15 days) to polypropylene microplastics (PP-MPs) at concentrations of 0.14 and 0.28 mg/L induced significant immunotoxic and oxidative stress in *C. gariepinus*. The observed effects included disruptions in leucocyte profiles, immunological biomarkers, and antioxidant enzyme activities (GST, SOD, CAT), along with elevated lipid peroxidation (MDA levels). Histopathological examinations revealed structural damage in key immune organs particularly the head kidney and spleen with an increase in melanomacrophage populations, indicating immune system activation and tissue stress. Co-treatment with *Spirulina* (200 mg/L) provided partial protection against the toxic effects of PP-MPs, reflected by improvements in some biochemical and immunological parameters. However, the degree of amelioration suggests that a higher *Spirulina* dose may be necessary for significant protective effects. Following a 45-day recovery period under normal conditions, there was substantial improvement in antioxidant and immune parameters, although complete histological restoration of immune organs lagged behind. This indicates that while physiological functions may recover relatively quickly, structural repair of immune tissues requires a longer time frame. The findings highlight the environmental and biological risks posed by PP-MPs, particularly those originating from widely used consumer items such as disposable paper cups. The evident toxicological impacts on fish underline the urgent need for stricter control of plastic waste discharge and more research into effective bioremediation strategies.

## Data Availability

All relevant raw data will be freely available from the authors.
